# Analysis of Plasma Epstein–Barr Virus DNA and Clinical Outcomes to Pembrolizumab or Chemotherapy in Recurrent/Metastatic Nasopharyngeal Cancer in KEYNOTE‐122

**DOI:** 10.1002/cam4.71496

**Published:** 2026-02-03

**Authors:** Anthony T. C. Chan, Victor Ho Fun Lee, Ruey‐Long Hong, Myung‐Ju Ahn, Wan Qin Chong, Anna Spreafico, Sung‐Bae Kim, Gwo Fuang Ho, Priscilla B. Caguioa, Nuttapong Ngamphaiboon, Ramona F. Swaby, Bo Wei, Andrea L. Webber, John Kang, Burak Gumuscu, Jianda Yuan, Lillian L. Siu

**Affiliations:** ^1^ State Key Laboratory in Translational Oncology, School of Biomedical Sciences, Sir YK Pao Centre for Cancer The Chinese University of Hong Kong Hong Kong China; ^2^ The University of Hong Kong Hong Kong China; ^3^ National Taiwan University Cancer Center Taipei Taiwan; ^4^ Samsung Medical Center, Sungkyunkwan University School of Medicine Seoul Republic of Korea; ^5^ National University Cancer Institute Singapore Singapore; ^6^ Princess Margaret Cancer Centre University of Toronto Toronto Ontario Canada; ^7^ Asan Medical Center University of Ulsan College of Medicine Seoul Republic of Korea; ^8^ Faculty of Medicine Universiti Malaya Kuala Lumpur Malaysia; ^9^ St. Luke's Medical Center University of Santo Tomas Faculty of Medicine and Surgery Manila Philippines; ^10^ Faculty of Medicine, Ramathibodi Hospital Mahidol University Bangkok Thailand; ^11^ Merck & Co. Inc. Rahway New Jersey USA

**Keywords:** biomarkers, chemotherapy, Epstein–Barr virus, nasopharyngeal carcinoma, pembrolizumab

## Abstract

**Background:**

Plasma Epstein–Barr virus (EBV) DNA has clinical utility for prognosis, recurrence, surveillance, and treatment response in nasopharyngeal carcinoma (NPC). This exploratory analysis evaluated associations between plasma EBV DNA load and clinical outcomes in participants treated with pembrolizumab or chemotherapy in the phase 3 KEYNOTE‐122 trial (NCT02611960).

**Methods:**

Participants with platinum‐pretreated, histologically confirmed, EBV‐positive, recurrent/metastatic NPC were randomly assigned (1:1) to pembrolizumab 200 mg intravenously every 3 weeks (≤ 35 cycles) or standard of care (SOC; investigator's choice of capecitabine, gemcitabine, or docetaxel). Associations between baseline plasma EBV DNA load as a continuous variable and plasma EBV DNA fold change at cycle 2 day 1 (C2D1), with clinical outcomes (progression‐free survival [PFS], overall survival [OS], and objective response rate [ORR]) were evaluated within each treatment arm. Nominal significance was prespecified at 0.05 for 1‐sided *p* values.

**Results:**

Of 228 treated participants, 215 (94.3%) had evaluable baseline plasma EBV DNA load data (pembrolizumab, 111; SOC, 104). Baseline plasma EBV DNA load was negatively associated with PFS and OS for pembrolizumab and SOC (both *p* < 0.005) but not ORR (*p* = 0.105, pembrolizumab; *p* = 0.473, SOC). Larger decreases in plasma EBV DNA load at C2D1 relative to baseline were associated with improved PFS, OS, and ORR for pembrolizumab and SOC (*p* ≤ 0.005).

**Conclusions:**

Higher baseline plasma EBV DNA load was negatively associated with outcomes in participants with NPC treated with pembrolizumab or SOC. These findings provide additional support for plasma EBV DNA as a prognostic biomarker for NPC.

**Trial Registration:**

ClinicalTrials.gov, NCT02611960

## 1 Introduction

First‐line standard‐of‐care (SOC) treatment for recurrent/metastatic nasopharyngeal carcinoma (NPC) is cisplatin plus gemcitabine; however, the 12‐month progression‐free survival (PFS) rate is only 20% [1, 2]. Recently, the anti‐programmed cell death protein 1 (PD‐1) inhibitor toripalimab in combination with cisplatin and gemcitabine was approved by the US Food and Drug Administration as a first‐line treatment option for this patient population [3, 4]. Despite advances in the treatment landscape, many patients experience disease recurrence, and it is important to identify patient groups who would be most likely to benefit from anti‐PD‐1 treatment.

Epstein–Barr virus (EBV) infection is strongly associated with the development of non‐keratinizing NPC [5, 6]. Plasma EBV DNA is established as a sensitive and specific biomarker for prognosis, surveillance of recurrence, and treatment response in NPC [5, 7, 8, 9, 10]. A prospective study reported detectable concentrations of plasma EBV DNA in 95% of participants with advanced NPC [11]. Furthermore, overall survival (OS) and relapse‐free survival were significantly shorter among participants with higher pretreatment and persistently detectable plasma EBV DNA concentrations compared with those who had lower concentrations of pretreatment EBV DNA and undetectable EBV DNA after radiotherapy [11]. Results from preclinical studies suggest that targeting the PD‐1/programmed cell death ligand 1 (PD‐L1) pathway can restore anti‐EBV tumor antigen–specific T‐cell immunity in patients with NPC [12, 13].

In the open‐label, randomized, phase 3 KEYNOTE‐122 trial, pembrolizumab versus chemotherapy was evaluated in participants with recurrent/metastatic NPC who were previously treated with platinum chemotherapy [14]. Although the study did not significantly improve the primary end point of overall survival (OS; hazard ratio [HR], 0.90 [95% CI, 0.67–1.19; *p* = 0.23]) for pembrolizumab versus chemotherapy, identifying clinically relevant biomarkers could help determine how patients may respond to anti–PD‐1 treatment. Here we explore the association of plasma EBV DNA load with clinical outcomes in participants with recurrent/metastatic NPC who received pembrolizumab and chemotherapy in KEYNOTE‐122.

## 2 Materials and Methods

### 2.1 Study Design and Participants

KEYNOTE‐122 was a randomized, open‐label, phase 3 trial conducted across 29 sites globally. Detailed study design and trial methods have been previously reported [14]. Briefly, adults aged ≥ 18 years with histologically confirmed, nonkeratinizing, differentiated (World Health Organization [WHO] type II) or undifferentiated (WHO type III), EBV‐positive (per EBV‐encoded small RNA in situ hybridization assay), recurrent/metastatic NPC, an Eastern Cooperative Oncology Group performance status (ECOG PS) of 0 or 1, and who previously received ≥ 1 platinum‐based chemotherapy for recurrent/metastatic disease were eligible to enroll. The representativeness of the trial participants is described in Table S1.

### 2.2 Procedures

Participants were randomly assigned in a 1:1 ratio to receive pembrolizumab 200 mg intravenously every 3 weeks for up to 35 cycles or investigator's choice of SOC chemotherapy with capecitabine 1000 mg/m^2^ (or 1250 mg/m^2^ from cycle 2 based on tolerability and local practices) orally twice daily on days 1–14 per 3‐week cycle, gemcitabine 1250 mg/m^2^ intravenously on days 1 and 8 per 3‐week cycle, or docetaxel 75 mg/m^2^ intravenously on day 1 per 3‐week cycle. Treatment continued until confirmed radiographic disease progression, unacceptable toxicity, investigator or participant decision to withdraw, or the maximum number of cycles was reached.

The study was conducted in accordance with principles of Clinical Practice and was approved by the appropriate institutional review boards and regulatory agencies from the following sites: British Columbia Cancer, Princess Margaret Cancer Centre, Prince of Wales Hospital, Princess Margaret Hospital, Queen Mary Hospital, Queen Elizabeth Hospital, Gleneagles Hospital Penang, University Malaya Medical Centre, Sarawak General Hospital, St. Luke's Medical Center, Davao Doctors Hospital, Cardinal Santos Medical Center, Cebu Doctors University Hospital, National University Hospital, National Cancer Centre Singapore, Samsung Medical Center, Asan Medical Center, National Cancer Center, National Taiwan University Hospital, National Cheng Kung University Hospital, Ramathibodi Hospital, Faculty of Medicine Siriraj Hospital, Chulalongkorn Hospital, University of California San Francisco, Yale Cancer Center, Northwestern Medicine, and City of Hope. All participants provided written informed consent.

### 2.3 Outcomes and Assessments

The primary end point of the KEYNOTE‐122 study was OS. The secondary end points were PFS, objective response rate (ORR), duration of response (DOR), and 6‐ and 12‐month PFS rates, all per RECIST v1.1 by blinded independent central review (BICR); 12‐ and 24‐month OS rates; and safety. In this exploratory analysis, the primary objectives were to determine an association between baseline plasma EBV DNA load and clinical outcomes (ORR, PFS, and OS) within each treatment arm; an association between plasma EBV load fold change from baseline at cycle 2 day 1 (C2D1), after baseline adjustment; and clinical outcomes within each treatment arm. The secondary objectives were to evaluate the association between baseline plasma EBV DNA load and tumor burden (measured by the sum of the longest diameter of the target lesions at baseline) in predicting clinical outcomes (PFS, OS) and the clinical utility of baseline plasma EBV DNA subgroups categorized by EBV DNA load.

ORR was defined as the best confirmed response of complete response or partial response per RECIST v1.1 by BICR. PFS was defined as the time from the start of treatment to the first documented evidence of disease progression per RECIST v1.1 by BICR or death due to any cause, whichever occurred first. OS was defined as the time from the start of treatment to death due to any cause.

Plasma EBV DNA was collected weekly during cycle 1 and then up to 3 days prior to dosing at all subsequent cycles. Analyses were performed by a central laboratory and plasma EBV DNA load was determined using a high‐sensitivity quantitative polymerase chain reaction assay (qPCR) that targets the repetitive BamHI‐W sequences [15]. The sequence of the primers and probe of the qPCR assay was published previously [15] with the modification of the quencher dyes of the probe as /56‐FAM/CACACACTA/ZEN/CACACACCCACCCGTCTC/3IABkFQ. Before DNA extraction, the plasma samples were thawed and then centrifuged at 20,000 × *g* for 5 min to pellet all cells and cell debris. DNA was extracted from 500 μL of the plasma supernatant using the NUCLISENS easyMAG system (BioMerieux, Marcy‐l'Étoile, France). PCR amplification was performed with the LightCycler 480 Probes Master mix (Roche, Basel, Switzerland) on the C480 II instrument (Roche) with the PCR conditions of 95°C for 10 min, 45 cycles of 95°C for 15 s, and 60°C for 1 min. For each plasma DNA sample, triplicate PCR reactions were performed and the plasma EBV DNA load was determined by a PCR standard curve established with serial dilutions of gBlock DNA containing the target region. The limit of detection of the assay was 20 copies of BamHI‐W repeat per mL of plasma, with linear dynamic range of 78 to 1 × 10^9^ copies/mL.

### 2.4 Statistical Analysis

This exploratory analysis included all participants who received ≥ 1 dose of study treatment and had evaluable plasma EBV DNA load data at baseline. The associations between plasma EBV DNA load and clinical outcomes were evaluated using the logistic regression model and area under the receiver operating characteristic curve (AUROC) with 95% confidence intervals (CIs) for ORR and Cox proportional hazards regression for PFS and OS, adjusting for ECOG PS. Plasma EBV DNA load was assessed as a continuous variable. Nominal significance was prespecified at 0.05 for 1‐sided *p* values without multiplicity adjustment. The association between plasma EBV DNA load and tumor burden was evaluated using the Spearman correlation coefficient.

The clinical utility of EBV was assessed in baseline plasma EBV DNA load‐high and baseline plasma EBV DNA load‐low subgroups, and in plasma EBV DNA load fold change‐high and plasma EBV DNA load fold change‐low subgroups, categorized by the median values. The Miettinen–Nurminen method was used to compare ORR between the treatment groups. PFS and OS were estimated using the Kaplan–Meier method. Participants without documented progression or death were censored at the date of the last adequate assessment for PFS analyses. Participants without documented death at the time of OS analysis were censored at the date of last follow‐up.

## 3 Results

### 3.1 Participants

Between May 5, 2016, and May 28, 2018, 233 participants were randomly assigned to receive pembrolizumab or SOC chemotherapy. The median follow‐up, defined as time from randomization to data cutoff (November 30, 2020), was 45.1 months (interquartile range, 39.0–48.8). Of the 228 participants who received ≥ 1 dose of study treatment, plasma EBV DNA data were available for 215 participants (pembrolizumab, *n* = 111; chemotherapy, *n* = 104); 13 participants were missing either baseline or C2D1 samples. Baseline characteristics in the EBV‐evaluable population were comparable between treatment groups (Table 1). Median baseline plasma EBV DNA load was 16.2 log_2_ copies/mL in the pembrolizumab arm and 15.2 log_2_ copies/mL in the chemotherapy arm (median of 15.8 log_2_ copies/mL across arms).

**TABLE 1:** Baseline characteristics of the NPC participant population with evaluable plasma EBV DNA enrolled in the KEYNOTE‐122 study.

	Pembrolizumab *n* = 111	SOC *n* = 104
Age, mean (range), years	50.7 (21–76)	53.3 (23–78)
Sex, *n* (%)		
Male	92 (82.8)	86 (82.7)
Female	19 (17.1)	18 (17.3)
ECOG PS, *n* (%)		
0	34 (30.6)	32 (30.8)
1	77 (69.4)	72 (69.2)
Overall cancer stage, *n* (%)		
I	1 (0.9)	0
II	1 (0.9)	0
III	1 (0.9)	3 (2.9)
IV	108 (97.3)	101 (97.1)
Liver metastases, *n* (%)		
Yes	50 (45.0)	49 (47.1)
No	61 (55.0)	55 (52.9)
EBV DNA, Log_2_ copies/mL, median	16.2	15.2

Abbreviations: EBV, Epstein–Barr virus; ECOG PS, Eastern Cooperative Oncology Group performance status; SOC, standard of care.

### 3.2 Association Between Baseline Plasma EBV DNA Load and Clinical Outcomes

**FIGURE 1:**
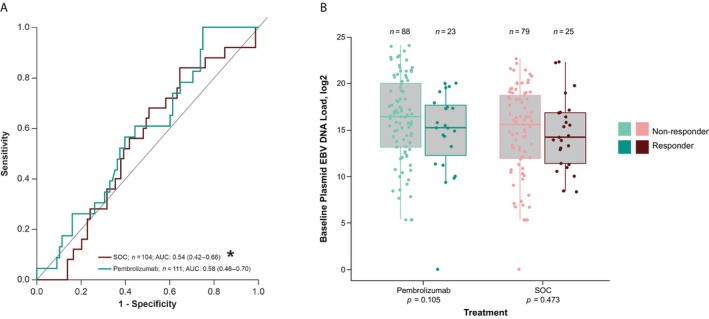
(A) Receiver operating characteristic curves of baseline plasma EBV DNA^a^ in predicting clinical outcome to pembrolizumab and SOC and (B) association between continuous baseline plasma EBV DNA load and ORR. AUC, area under the curve; AUROC, area under the receiver operating characteristic curve; EBV, Epstein–Barr virus; ORR, objective response rate; SOC, standard of care. ^a^In log_2_ scale. *AUROC calculated in the direction based on the hypothesized direction of effect.

Higher baseline plasma EBV DNA load as a continuous variable was significantly negatively associated with PFS and OS for pembrolizumab (both, nominal *p* < 0.0001) and SOC (nominal *p* = 0.001 and *p* < 0.0001, respectively), but not with ORR (nominal *p* = 0.105, pembrolizumab; nominal *p* = 0.473, SOC). The AUROC for discriminating plasma EBV DNA load as a predictor of objective response was 0.58 (95% CI, 0.46–0.70) for pembrolizumab and 0.54 (95% CI, 0.42–0.66) for SOC (Figure 1A). In both treatment arms, plasma EBV DNA was higher in nonresponders than in responders (Figure 1B).

### 3.3 Association Between Plasma EBV DNA Load Fold Change at C2D1 and Clinical Outcomes

**FIGURE 2:**
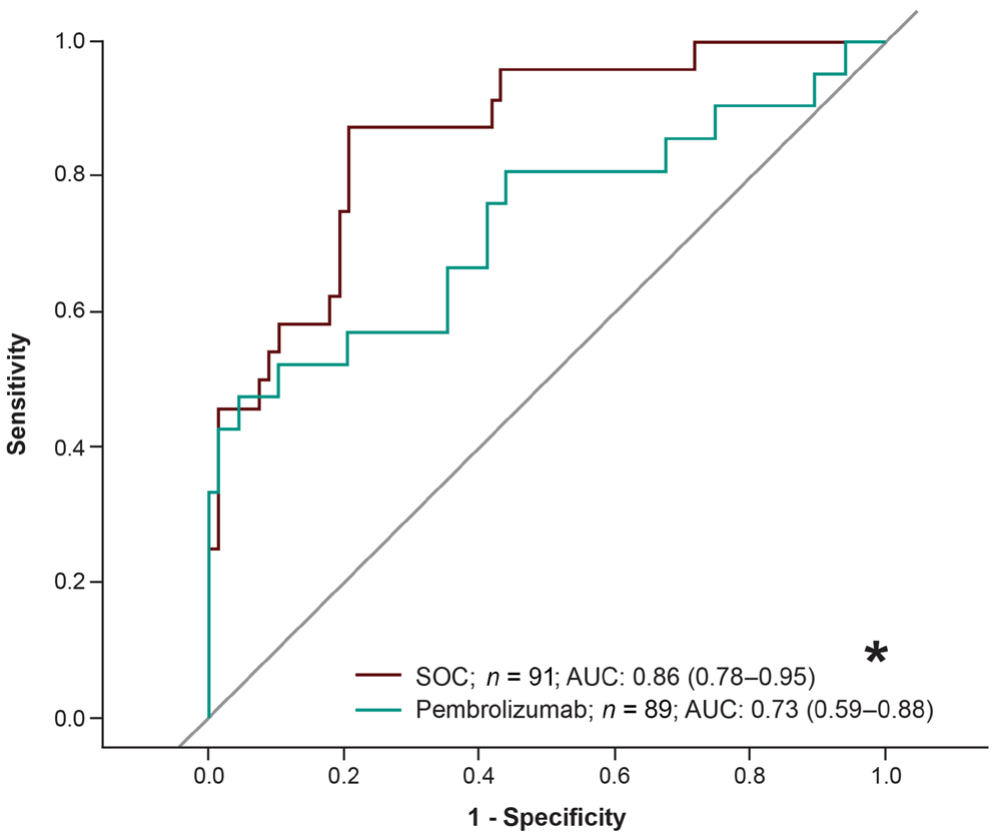
Receiver operating characteristic curves of fold change of plasma EBV DNA^a^ from baseline at C2D1 in predicting clinical outcomes for treatment with pembrolizumab and SOC. AUC, area under the curve; AUROC, area under the receiver operating curve; C2D1, cycle 2 day 1; EBV, Epstein–Barr virus; SOC, standard of care. ^a^In log_2_ scale. *AUROC calculated in the direction based on the hypothesized direction of effect.

Larger decreases in plasma EBV DNA load at C2D1 relative to baseline were significantly associated with better ORR, PFS, and OS for pembrolizumab (nominal *p* < 0.001, nominal *p* < 0.001, and nominal *p* < 0.01, respectively) and for SOC (all, nominal *p* < 0.0001). The AUROC for discriminating plasma EBV DNA load fold change at C2D1 as a predictor of objective response was 0.73 (95% CI, 0.59–0.88) for pembrolizumab and 0.86 (95% CI, 0.78–0.95) for SOC (Figure 2). In both treatment arms, the plasma EBV DNA load fold change between responders and nonresponders increased over time (Figure S1).

### 3.4 Association Between Baseline Plasma EBV DNA Load and Tumor Burden in Predicting Clinical Outcomes

**FIGURE 3:**
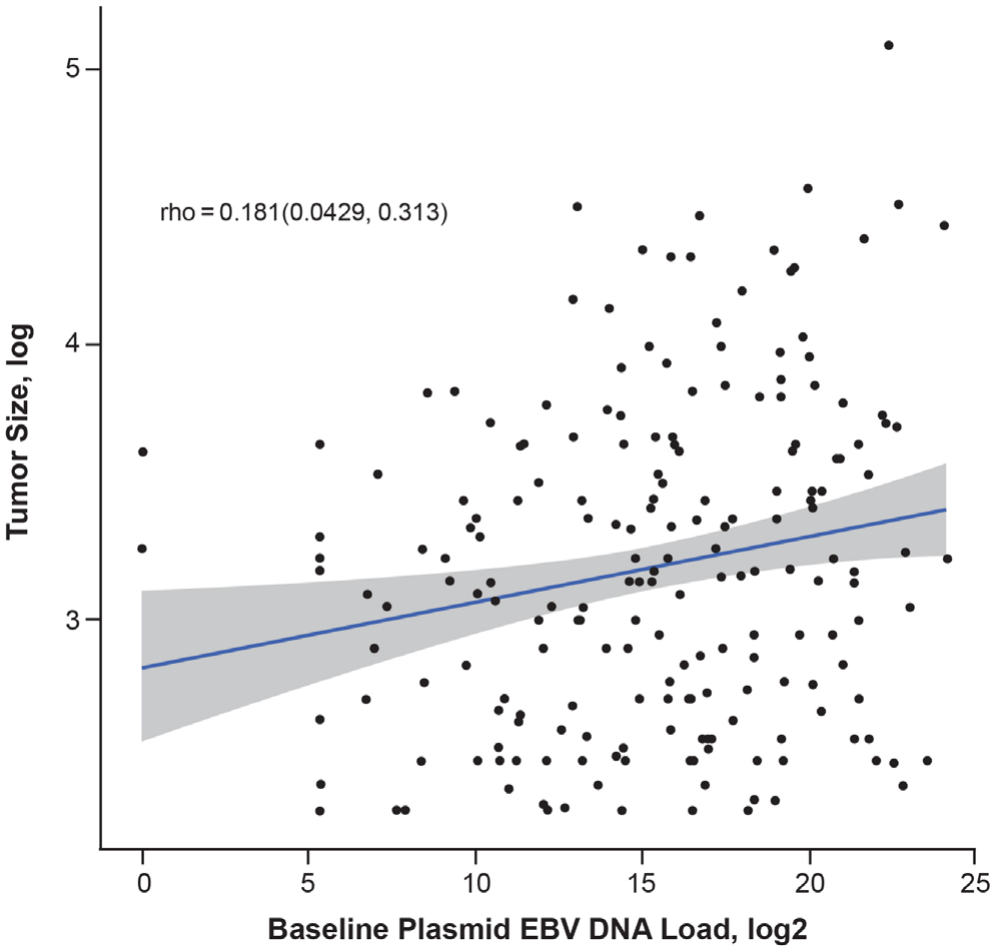
Correlation between baseline plasma EBV DNA load and tumor size measurement in both arms prior to treatment. EBV, Epstein–Barr virus.

There was a weak association between plasma EBV DNA load and radiographic tumor burden at baseline (*ρ* = 0.18; Figure 3). Baseline plasma EBV DNA load was numerically more predictive of OS and PFS than tumor volume in both treatment arms (Table 2).

**TABLE 2:** Comparison of the association between clinical outcomes and baseline plasma EBV DNA load and radiographic tumor burden.

	Pembrolizumab		SOC	
	Tumor volume	EBV DNA load	Tumor volume	EBV DNA load
PFS, C index (95% CI)	0.42 (0.35–0.49)	0.67 (0.60–0.74)	0.54 (0.46–0.62)	0.64 (0.56–0.71)
OS, C index (95% CI)	0.47 (0.41–0.54)	0.71 (0.64–0.77)	0.58 (0.51–0.65)	0.67 (0.60–0.73)

Abbreviations: C index, concordance index; EBV, Epstein–Barr virus; OS, overall survival; PFS, progression‐free survival; SOC, standard of care.

### 3.5 Outcomes by Baseline Plasma EBV DNA Load Cutoffs

**FIGURE 4:**
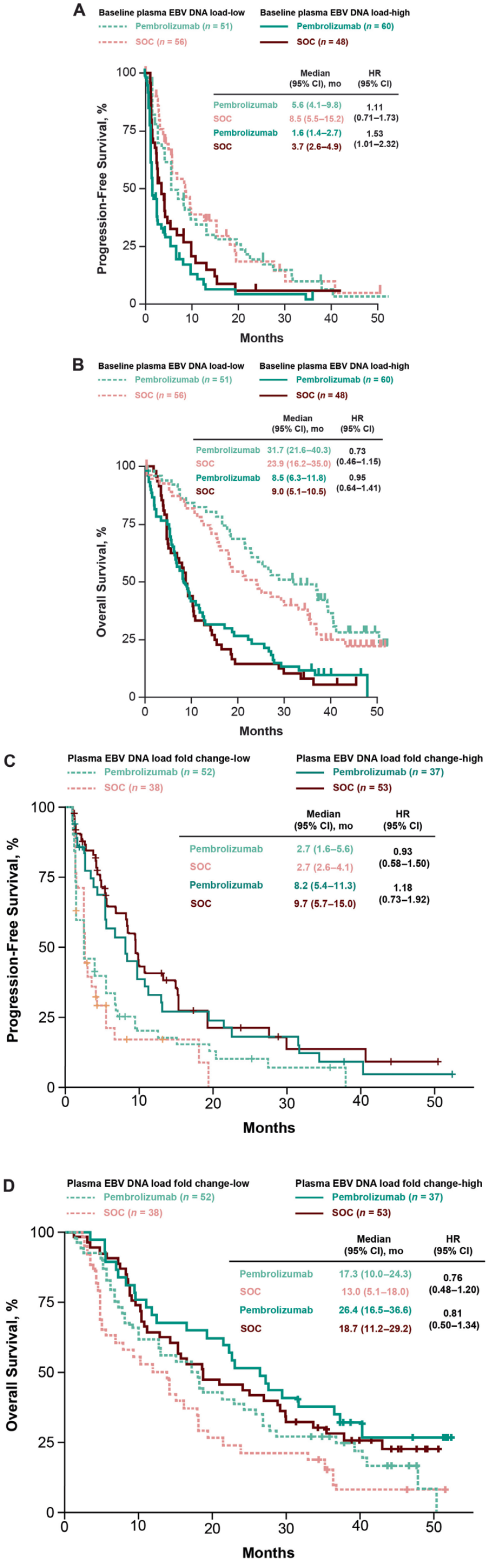
(A) PFS and (B) OS by baseline plasma EBV DNA load subgroup and (C) PFS and (D) OS by plasma EBV DNA load fold change in each treatment arm. EBV, Epstein–Barr virus; HR, hazard ratio; OS, overall survival; PFS, progression‐free survival; SOC, standard of care. Median, 15.8 log_2_ copies/mL; median, −0.7649 log_2_ copies/mL.

Using the median value of baseline plasma EBV DNA load of 15.8 log_2_ copies/mL as a cutoff, 51 participants in the pembrolizumab group had baseline plasma EBV DNA load‐low tumors and 60 participants had baseline plasma EBV DNA load‐high tumors; 56 participants in the SOC group had baseline plasma EBV DNA load‐low tumors and 48 participants had baseline plasma EBV DNA load‐high tumors. For participants in the baseline plasma EBV DNA load‐low group, ORR was 25.5% for pembrolizumab and 28.6% for SOC (difference, −3.1 [95% CI, −19.8 to 14.0]). Median PFS was 5.6 months for pembrolizumab and 8.5 months for SOC (HR, 1.11 [95% CI, 0.71–1.73]; Figure 4A). Median OS was 31.7 months for pembrolizumab and 23.9 months for SOC (HR, 0.73 [95% CI, 0.46–1.15]) (Figure 4B). For participants in the baseline plasma EBV DNA load‐high group, ORR was 16.7% for pembrolizumab and 18.8% for SOC (difference, −2.1 [95% CI, −17.5 to 12.4]). Median PFS was 1.6 months for pembrolizumab and 3.7 months for SOC (HR, 1.53 [95% CI, 1.01–2.32]). Median OS was 8.5 months for pembrolizumab and 9.0 months for SOC (HR, 0.95 [95% CI, 0.64–1.41]). Compared with baseline plasma EBV DNA load‐high, PFS favored participants with baseline plasma EBV DNA load‐low in the pembrolizumab group (HR, 0.44 [95% CI, 0.29–0.67]) and in the SOC group (HR, 0.56 [95% CI, 0.35–0.88]). OS also favored participants with baseline plasma EBV DNA load‐low versus plasma EBV DNA load‐high in the pembrolizumab group (HR, 0.32 [95% CI, 0.20–0.50]) and in the SOC group (HR, 0.38 [95% CI, 0.25–0.59]).

### 3.6 Outcomes by Plasma EBV DNA Load Fold Change at C2D1

Using the median fold change from baseline to C2D1 value of −0.7649 log_2_ copies/mL as a cutoff, 52 participants in the pembrolizumab group had plasma EBV DNA load fold change‐low tumors and 37 participants had plasma EBV DNA load fold change‐high tumors; 38 participants in the SOC group had plasma EBV DNA load fold change‐low tumors and 53 participants had plasma EBV DNA load fold change‐high tumors. For participants in the plasma EBV DNA load fold change‐low group, median PFS was 2.7 months for pembrolizumab and 2.7 months for SOC (HR, 0.93 [95% CI, 0.58–1.50]; Figure 4C). Median OS was 17.3 months for pembrolizumab and 13.0 months for SOC (HR, 0.76 [95% CI, 0.48–1.20]) (Figure 4D). For participants in the plasma EBV DNA load fold change‐high group, median PFS was 8.2 months for pembrolizumab and 9.7 months for SOC (HR, 1.18 [95% CI, 0.73–1.92]). Median OS was 26.4 months for pembrolizumab and 18.7 months for SOC (HR, 0.81 [95% CI, 0.50–1.34]). Compared with plasma EBV DNA load fold change‐high, PFS did not favor participants with EBV DNA load fold change‐low in the pembrolizumab group (HR, 1.82 [95% CI, 1.14–2.94]) or in the SOC group (HR, 2.78 [95% CI, 1.67–4.76]). OS did not favor participants with plasma EBV DNA load fold change‐low versus plasma EBV DNA load fold change‐high in the pembrolizumab group (HR, 1.64 [95% CI, 1.00–2.70]) or in the SOC group (HR, 1.67 [95% CI, 1.05–2.63]).

## 4 Discussion

This exploratory analysis of KEYNOTE‐122 showed that higher baseline EBV DNA load was negatively associated with PFS and OS, but not ORR, in participants with platinum‐pretreated recurrent/metastatic NPC who received pembrolizumab or SOC chemotherapy. Independent of the baseline association, a larger decrease in plasma EBV DNA load at C2D1 relative to baseline was associated with better clinical outcomes (ORR, PFS, OS) with either treatment. OS was longest in participants who received pembrolizumab and had a high plasma EBV DNA fold change (median OS, 26.4 months). OS was similar between participants in the pembrolizumab group with low plasma EBV DNA load fold change (median OS, 17.3 months) and those in the SOC chemotherapy group with high plasma EBV DNA fold change (median OS, 18.7 months). OS was lowest in participants who received SOC chemotherapy and had a low plasma EBV DNA load fold change (median OS, 13.0 months). Results suggest participants with a larger plasma EBV DNA load fold change from baseline may benefit from pembrolizumab.

Baseline plasma EBV DNA load was numerically more predictive of outcomes than tumor volume for both pembrolizumab and SOC. Objective response rates were similar for pembrolizumab and SOC in each of the baseline plasma EBV DNA subgroups categorized by EBV DNA load.

The observation that plasma EBV DNA load is associated with clinical outcomes in this study is consistent with findings from other studies evaluating anti–PD‐L1 agents in NPC. In a meta‐analysis of patients with NPC receiving immune checkpoint inhibitors (ICIs), lower baseline plasma EBV DNA load, decreased plasma EBV DNA load during treatment, and higher PD‐L1 expression were predictive of some clinical benefit to ICI treatment [16]. Trends observed when evaluating baseline plasma EBV DNA load are consistent with those reported for participants with recurrent/metastatic NPC treated with the anti–PD‐L1 treatment toripalimab from the POLARIS‐02 study [17]. Lower pretreatment EBV titers (< 10,000 IU/mL) had numerically higher ORR (26.7%) compared with titers of ≥ 10,000 IU/mL (15.4%); however, the difference was not statistically significant (*p* = 0.088) [17]. In a prospective analysis of the POLARIS‐02 trial, higher baseline EBV DNA titer was associated with shorter median PFS (HR, 1.70, 95% CI, 1.21–2.40; *p* = 0.002) and median OS (HR, 1.88 [95% CI, 1.22–2.89]; *p* = 0.004) compared with low baseline EBV DNA titer [18].

In the KEYNOTE‐028 trial, a correlation between plasma EBV DNA levels with clinical outcomes to pembrolizumab was observed in 4 participants with recurrent/metastatic NPC who had available serial EBV DNA samples [19]. In KEYNOTE‐028, plasma EBV DNA was analyzed by a certified laboratory using RealStar EBV PCR Kit (Altona Diagnostics, Hamburg, Germany) and ABI 7500 (Applied Biosystems, Waltham, Massachusetts). Of the 2 participants with partial responses, 1 received ≥ 23 cycles of pembrolizumab and had plasma EBV DNA under 1000 copies/mL throughout treatment, and the other experienced tumor progression and had a detectable increase in plasma EBV DNA 6 weeks prior to radiographic detection.

Similar trends were observed when evaluating plasma EBV DNA changes relative to baseline levels. Early clearance of plasma EBV DNA was shown to be associated with the response rate of camrelizumab in combination with chemotherapy [20]. In the POLARIS‐02 trial, participants with ≥ 50% decrease in plasma EBV DNA titers on day 28 had significantly better ORR (48.3%) than participants with < 50% decrease (5.7%; *p* = 0.0001) [17]. In the JUPITER‐02 study of toripalimab plus chemotherapy versus placebo plus chemotherapy in participants with recurrent/metastatic NPC, significantly more participants in the toripalimab arm had their EBV DNA copy number decrease to undetectable levels compared to the placebo arm (96.3% vs. 84.5%; *p* = 0.004) [21]. Fewer participants in the toripalimab arm had EBV DNA rebound than in the placebo arm.

Interestingly, we observed a higher AUC in the SOC group compared with the pembrolizumab group, indicating that the postbaseline EBV DNA change with SOC was more predictive of ORR than pembrolizumab. Chemotherapy tends to have a quicker response time than immunotherapy because chemotherapy directly targets rapidly dividing cells, leading to immediate tumor shrinkage, while immunotherapy stimulates a person's immune system to recognize and fight cancer cells and can take several weeks to months to develop a measurable response. In the initial stages of immunotherapy, there may also be potential “pseudo‐progression” due to the influx of immune cells [22]. These differences may help explain why the postbaseline change in the SOC group with chemotherapy treatment would be more predictive of ORR than pembrolizumab at C2D1.

Despite the lack of correlation between plasma EBV DNA load and tumor burden in our analysis, a linear relationship between pretreatment plasma EBV DNA and tumor burden has been reported in patients with NPC [23, 24, 25]. The relationship is still poorly understood, however, and the differences we observed may be due to factors that affect the rate of release and clearance of plasma EBV DNA [24]. Furthermore, the difference in tumor assessments may have contributed to the lack of correlation observed in our study. In this study, tumor burden was measured by the sum of the longest diameter of the target lesions at baseline compared with the study by Ma et al. [23], which used CT or MRI scans for tumor volume calculation.

This analysis is limited by its exploratory nature and the use of 1‐sided *p* values without multiplicity adjustment. Further, the small sample size limits the ability to draw definitive conclusions, and the small number of available studies evaluating plasma EBV DNA in patients who received anti–PD‐L1 treatment limits comparability.

## 5 Conclusion

This exploratory analysis from KEYNOTE‐122 showed plasma EBV DNA load was associated with clinical outcomes in participants with platinum‐pretreated recurrent/metastatic NPC who received pembrolizumab or SOC. While definitive conclusions are limited, these findings provide additional support for plasma EBV DNA load as a prognostic biomarker for NPC and could guide clinical treatment decisions for NPC.

## Author Contributions

**Anthony T. C. Chan:** conceptualization (equal), investigation (equal), writing – original draft (equal), writing – review and editing (equal). **Victor Ho Fun Lee:** investigation (equal), writing – review and editing (equal). **Ruey‐Long Hong:** investigation (equal), writing – review and editing (equal). **Myung‐Ju Ahn:** investigation (equal), writing – review and editing (equal). **Wan Qin Chong:** investigation (equal), writing – review and editing (equal). **Anna Spreafico:** investigation (equal), writing – review and editing (equal). **Sung‐Bae Kim:** investigation (equal), writing – review and editing (equal). **Gwo Fuang Ho:** investigation (equal), writing – review and editing (equal). **Priscilla B. Caguioa:** investigation (equal), writing – review and editing (equal). **Nuttapong Ngamphaiboon:** investigation (equal), writing – review and editing (equal). **Ramona F. Swaby:** conceptualization (equal), formal analysis (equal), investigation (equal), writing – review and editing (equal). **Bo Wei:** data curation (lead), formal analysis (equal), methodology (lead), validation (lead), writing – review and editing (equal). **Andrea L. Webber:** conceptualization (equal), investigation (equal), writing – review and editing (equal). **John Kang:** investigation (equal), writing – original draft (equal), writing – review and editing (equal). **Burak Gumuscu:** investigation (equal), writing – review and editing (equal). **Jianda Yuan:** conceptualization (equal), investigation (equal), writing – review and editing (equal). **Lillian L. Siu:** project administration (lead), writing – review and editing (equal).

## Funding

This study was supported by Merck Sharp & Dohme LLC, a subsidiary of Merck & Co. Inc., Rahway, NJ, USA.

## Conflicts of Interest

Anthony T. C. Chan reports receiving grants or contracts (institution) from MSD, Pfizer, Angene Biotechnology, Novartis, and NRG Oncology Foundation; consulting fees (personal) from MSD and Tessa Therapeutics Ltd.; payment or honoraria for lectures, presentations, speakers bureaus, manuscripts, or other education events from Springer and Wolters Kluwer; support for attending meetings and/or travel from Roche; and participation on a Data Safety Monitoring Board or Advisory Board for MSD and Tessa Therapeutics Ltd. Victor Ho Fun Lee reports receiving medical writing support for the present manuscript from Pfizer; research support grants from AstraZeneca, Pfizer, and Varian Medical Systems; honoraria from Amgen, Roche, Pfizer, AstraZeneca, Takeda, Novartis, MSD, Eli Lilly, Merck, Top Alliance, Boston Scientific, and Varian Medical Systems. Ruey‐Long Hong has no conflicts to disclose. Wan Qin Chong reports payment or honoraria for lectures, presentations, speakers bureaus, manuscripts, or other education events from MSD and Ipsen; support for attending meetings and/or travel from Merck and AstraZeneca; and participation on a Data Safety Monitoring Board or Advisory Board (institution and personal) for Merck and DiethelmKellerSiberHegner. Myung‐Ju Ahn reports receiving consulting fees (personal) from AstraZeneca, Roche, MSD, Merck, Takeda, ONO Pharmaceuticals, Novartis, Amgen, YUHAN, Alpha Pharmaceuticals, Daiichi Sankyo, Pfizer, Voronoi, and Genexin and payment or honoraria for lectures, presentations, speakers bureaus, manuscripts, or other education events from AstraZeneca, Roche, MSD, Merck, Takeda, ONO Pharmaceuticals, Novartis, Amgen, YUHAN, and Daiichi Sankyo. Anna Spreafico reports consulting fees for Advisory Board participation from Merck, Bristol Myers Squibb, and BeiGene; grants/research support for clinical trials (institution) from Novartis, Bristol Myers Squibb, Symphogen AstraZeneca/Medimmune, Merck, Bayer, Surface Oncology, Northern Biologics, Janssen Oncology/Johnson & Johnson, Roche, Regeneron, Alkermes, Array Biopharma/Pfizer, GSK, Oncorus, Treadwell, Amgen, Teva Therapeutics, Merus, and Alentis. Sung‐Bae Kim reports receiving grants or contracts from Novartis, DongKook Pharm Co., and Sanofi‐Adventis; consulting fees from Novartis, Lilly, ISU Abix, AstraZeneca, Dae Hwa Pharmaceutical Co Ltd., OBI Pharma, BeiGene, and Daiichi Sanyko; payment or honoraria for lectures, presentations, speakers bureaus, manuscripts, or other education events from Novartis, Lilly, Daiichi Sankyo, AstraZeneca, and OBI Pharma. Gwo Fuang Ho reports receiving grants or contracts (institution) from Eli Lilly, Regeneron Pharmaceuticals, MSD, AB Science, Astellas, Tessa Therapeutics, Roche, Arcus Bioscience, AstraZeneca, Pfizer, Janssen Research & Development, Mirati Therapeutics, Novartis, Amgen, and Boehringer Ingelheim; payment or honoraria for lectures, presentations, speakers bureaus, manuscripts, or other education events (personal) from MSD, Novartis, F. Hoffman‐La Roche AG, AstraZeneca, Boehringer Ingelheim, Pfizer, Merck & Co. Inc., Rahway, NJ, USA, and Eisai; support for attending meetings and/or travel (personal) from Ipsen, AstraZeneca, Bristol Myers Squibb, MSD, Regeneron Pharmaceuticals, Dr. Reddy's, Roche, Servier, Zullig Pharma, and Pfizer; participates on a Data Safety Monitoring Board or Advisory Board for MSD, Novartis, Roche, AstraZeneca, Boehringer Ingelheim, Pfizer, Astellas, and Takeda; and receipt of equipment from Pfizer, Novartis, Janssen Pharmaceuticals, Taiho, and Eli Lilly. Priscilla B. Caguioa reports receiving support for clinical trials from Regeneron, AstraZeneca, MSD; speakers bureau: Johnson & Johnson, Takeda, and AstraZeneca. Nuttapong Ngamphaiboon reports receiving support for the present manuscript from MSD; grants or contracts (institution) from MSD, Roche, BeiGene, RAPT Therapeutics, Boehringer Ingelheim, and AstraZeneca; payment or honoraria for lectures, presentations, speakers bureaus, manuscripts, or other education events from Roche, Eisai, MSD, Amgen, Merck, and Bristol Myers Squibb; support for attending meetings and/or travel from Roche, MSD, Amgen, Merck, and Eisai; and participation on a Data Safety Monitoring Board or Advisory Board for Roche, MSD, Eisai, BeiGene, Bristol Myers Squibb, Amgen, Servier, and Ascendant Biotech Corporation. Ramona F. Swaby reports receiving support for attending meetings and/or travel from Carisma Therapeutics and Genmab and owns stock or stock options in Carisma Therapeutics and Genmab. Bo Wei is an employee of Merck Sharp & Dohme LLC, a subsidiary of Merck & Co. Inc., Rahway, NJ, USA, and owns stock options in Merck & Co. Inc., Rahway, NJ, USA. Andrea L. Webber reports receiving support for the present manuscript and support for attending meetings and/or travel from Merck; is an employee of Merck Sharp & Dohme LLC, a subsidiary of Merck & Co. Inc., Rahway, NJ, USA; and owns stock or stock options in Merck & Co. Inc., Rahway, NJ, USA, and Organon. John Kang is an employee of Merck Sharp & Dohme LLC, a subsidiary of Merck & Co. Inc., Rahway, NJ, USA, and owns stock or stock options in Merck & Co. Inc., Rahway, NJ, USA. Burak Gumuscu is an employee of Merck Sharp & Dohme LLC, a subsidiary of Merck & Co. Inc., Rahway, NJ, USA, and owns stock or stock options in Merck & Co. Inc., Rahway, NJ, USA. Jianda Yuan is an employee of Merck Sharp & Dohme LLC, a subsidiary of Merck & Co. Inc., Rahway, NJ, USA, and owns stock or stock options in Merck & Co. Inc., Rahway, NJ, USA. Lillian L. Siu reports receiving clinical trial support (institution) from Merck, Bristol Myers Squibb, Roche/Genentech, GlaxoSmithKline, Novartis, Pfizer, AstraZeneca, Boehringer Ingelheim, Bayer, Amgen, Daiichi Sankyo, EMD Serono, 23Me, Astellas, AbbVie, Gilead, Incyte, Legochem Biosciences, Loxo/Lilly, Medicenna, and Takara Bio; consulting fees (personal) for consultation/scientific advisory board from Merck, AstraZeneca, Bristol Myers Squibb, Roche/Genentech, Voronoi, GlaxoSmithKline, Seattle Genetics, Arvinas, Navire, Relay Therapeutics, Daiichi Sankyo, Tubulis, Medicenna, LTZ Therapeutics, Marengo, Nerviano, Amgen, Pangea, Incyte, and Gilead; and reports spouse as co‐founder of Treadwell Therapeutics with stock ownership in Agios Therapeutics.

## Supporting information


**Table S1:** Representativeness of study participants*.
**Figure S1:** Plasma EBV DNA load fold change over time.

## Data Availability

Merck Sharp & Dohme LLC, a subsidiary of Merck & Co. Inc., Rahway, NJ, USA (MSD), is committed to providing qualified scientific researchers access to anonymized data and clinical study reports from the company's clinical trials for the purpose of conducting legitimate scientific research. MSD is also obligated to protect the rights and privacy of trial participants and, as such, has a procedure in place for evaluating and fulfilling requests for sharing company clinical trial data with qualified external scientific researchers. The MSD data sharing website (available at: https://externaldatasharing‐msd.com/) outlines the process and requirements for submitting a data request. Applications will be promptly assessed for completeness and policy compliance. Feasible requests will be reviewed by a committee of MSD subject matter experts to assess the scientific validity of the request and the qualifications of the requestors. In line with data privacy legislation, submitters of approved requests must enter into a standard data‐sharing agreement with MSD before data access is granted. Data will be made available for request after product approval in the United States and the European Union or after product development is discontinued. There are circumstances that may prevent MSD from sharing requested data, including country‐ or region‐specific regulations. If the request is declined, it will be communicated to the investigator. Access to genetic or exploratory biomarker data requires a detailed hypothesis‐driven statistical analysis plan that is collaboratively developed by the requestor and MSD subject matter experts; after approval of the statistical analysis plan and execution of a data‐sharing agreement, MSD will either perform the proposed analyses and share the results with the requestor or will construct biomarker covariates and add them to a file with clinical data that is uploaded to an analysis portal so that the requestor can perform the proposed analyses.
