# Supplementary material for: Analysis of Plasma Epstein–Barr Virus DNA and Clinical Outcomes to Pembrolizumab or Chemotherapy in Recurrent/Metastatic Nasopharyngeal Cancer in KEYNOTE‐122
Source: Cancer Med. 2026 Feb 3;15(2):e71496. doi: 10.1002/cam4.71496 (PMC12866814; doi:10.1002/cam4.71496)

Supplementary Appendix

Table of Contents

Supplementary Table 1. Representativeness of study participants 2

Supplementary Figure 1. Plasma EBV DNA load fold change over time 3

Supplementary Table 1. Representativeness of study participants*

| Disease, problem, or condition under investigation | Previously treated recurrent/metastatic nasopharyngeal cancer |
| --- | --- |
| Special considerations related to |  |
| Sex and gender | Globally, the age-standardized incidence rates were 3-fold higher in men than women.^1^ |
| Age | NPC predominately affects middle-aged individuals.^2^ |
| Race/ethnicity | Nonkeratinizing NPC is endemic in the Asian population.^1,2^ |
| Geography | The largest numbers of cases and deaths from NPC were estimated in Eastern Asia, where the greatest contributor to global NPC burden was China,^1^ suggesting genetic and environmental risk factors contribute to the development of NPC.^2^ |
| Other considerations | EBV infection is as an important risk factor, with a dose-response relationship between EBV antibody level and NPC risk.^1^ |
| Overall representativeness of this trial | The population attributable fraction for NPC incidence due to EBV infection has been estimated to be 85%.^3^ It is recommended to consider screening for EBV in nonkeratinizing or undifferentiated NPC.^1,2^ |

*Information for this table was collected by searching publications in PubMed, as well as publications by widely known periodic epidemiological studies Global Cancer Observatory (GLOBOCAN). A full reference list is available at the end of this Supplementary Appendix. Research questions answered include: What are the age, sex and gender, and race or ethnic group demographics of patients affected by NPC? What risk factors are associated with the disease? What is the geographic distribution of the disease?

1.Zhang Y, Rumgay H, Li M, et al. Nasopharyngeal Cancer Incidence and Mortality in 185 Countries in 2020 and the Projected Burden in 2040: Population-Based Global Epidemiological Profiling. JMIR Public Health Surveill. 2023;9:e49968.

2. Lam WKJ, King AD, Miller JA, et al. Recommendations for Epstein-Barr virus–based screening for nasopharyngeal cancer in high- and intermediate-risk regions. J of the Nat Can Inst.2023;115(4):355–364.

3. De Martel C, Georges D, Bray F, et al. Global burden of cancer attributable to infections in 2018: a worldwide incidence analysis. Lancet Glob Health. 2020;8(2):e180-e190.

Supplementary Figure 1. Plasma EBV DNA load fold change over time


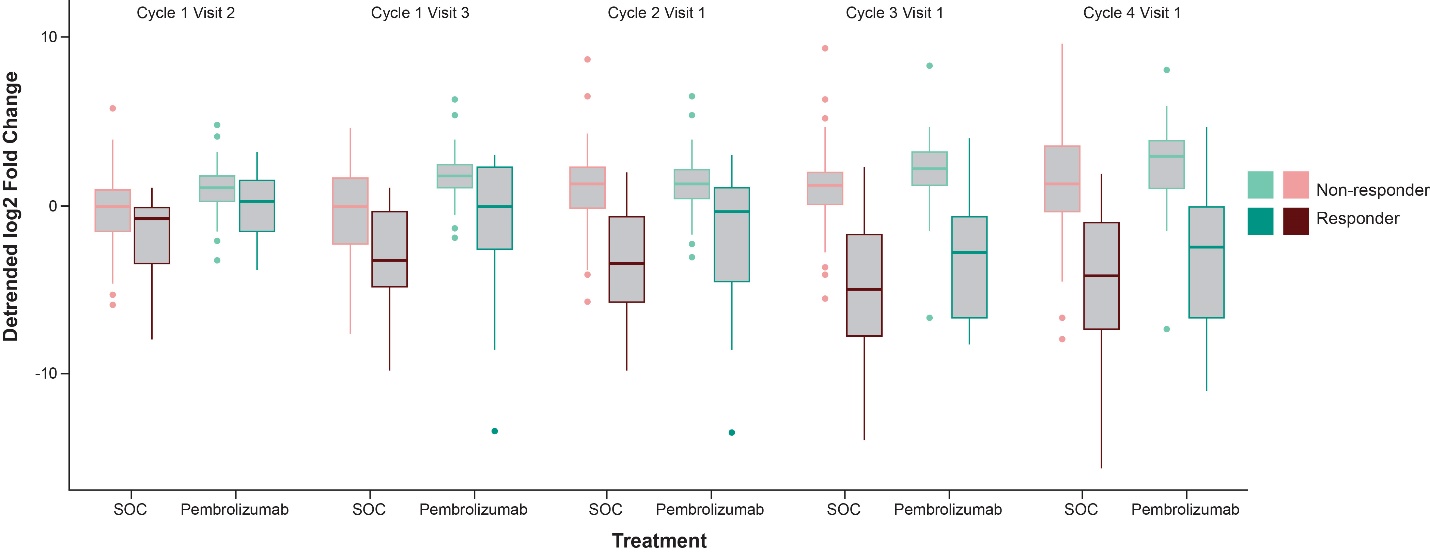

Supplement: Supplementary file 1 — Table S1: Representativeness of study participants*. Figure S1: Plasma EBV DNA load fold change over time. [file CAM4-15-e71496-s001.docx]
